# RETRACTED: Paladugu et al. Liraglutide Has Anti-Inflammatory and Anti-Amyloid Properties in Streptozotocin-Induced and 5xFAD Mouse Models of Alzheimer’s Disease. *Int. J. Mol. Sci.* 2021, *22*, 860

**DOI:** 10.3390/ijms27157042

**Published:** 2026-08-06

**Authors:** Leela Paladugu, Abeer Gharaibeh, Nivya Kolli, Cameron Learman, Tia C. Hall, Lixin Li, Julien Rossignol, Panchanan Maiti, Gary L. Dunbar

**Affiliations:** 1 Field Neurosciences Institute Laboratory for Restorative Neurology, Central Michigan University, Mount Pleasant, MI 48859, USA; 2 Program in Neuroscience, Central Michigan University, Mount Pleasant, MI 48859, USA; 3 Insight Research Center, Insight Institute of Neurosurgery and Neuroscience, Flint, MI 48607, USA; 4 Physician Assistant Program, Central Michigan University, Mount Pleasant, MI 48859, USA; 5 College of Medicine, Central Michigan University, Mount Pleasant, MI 48859, USA; 6 Field Neurosciences Institute, Ascension St. Mary’s, Saginaw, MI 48604, USA; 7 Department of Psychology, Central Michigan University, Mount Pleasant, MI 48859, USA; 8 College of Health and Human Services, Saginaw Valley State University, Saginaw, MI 48710, USA

The journal retracts the article entitled “Liraglutide Has Anti-Inflammatory and Anti-Amyloid Properties in Streptozotocin-Induced and 5xFAD Mouse Models of Alzheimer’s Disease” [1], cited above.

Following publication, concerns were raised regarding the integrity of certain figures presented in the article [1].

In accordance with the journal’s standard procedures, the Editorial Office and Editorial Board conducted an investigation. During this process, the histological data underlying the published findings could not be adequately verified. In response, the authors repeated the relevant experiments and provided additional data for assessment by the Editorial Board.

Following a review of the newly generated data and supporting information, the Editorial Board determined that the issues identified could not be satisfactorily resolved through a correction, as the revisions required to the published results were substantial.

Consequently, the Editorial Board has concluded that retraction of the article [1] is the most appropriate course of action, in accordance with MDPI’s retraction policy. The authors are encouraged to submit a revised manuscript incorporating the new data and findings as a new submission, which will be considered through the journal’s standard editorial and peer-review processes.

This retraction was approved by the Editor-in-Chief of the *International Journal of Molecular Sciences*.

The authors have been informed of this retraction. Gary L. Dunbar, Nivya Kolli, Lixin Li, Leela Paladugu and Abeer Gharaibeh have indicated their agreement with this decision. The other authors did not provide a comment on this decision.
